# Human dignity research in clinical practice – a systematic literature review

**DOI:** 10.1111/scs.12922

**Published:** 2020-10-26

**Authors:** Lillemor Lindwall, Vibeke Lohne

**Affiliations:** ^1^ University of Karlstad Karlstad Sweden; ^2^ Oslo Metropolitan University Oslo Norway

**Keywords:** caring science, human dignity, clinical practice, systematic literature review

## Abstract

**Background:**

This literature study describes caring science research on human dignity in different clinical practice. We already know a good deal about human dignity in nursing care but *how do patients, nurses, healthcare professionals and next of kin experience human dignity in clinical practice?*

**Aim:**

To summarise studies on human dignity to gain a deeper understanding of how it can be achieved in caring science research and to gain a broader understanding of the differences and similarities across caring contexts. The aim was also to gain a broader understanding of the differences and similarities of human dignity across different clinical practice.

**Method:**

The literature review re‐analysed 28 empirical studies on human dignity are experienced from acute, psychiatric, elderly and rehabilitation care. The data analysis strategy was conducted in a systematic and critical way and consisted of a five‐step method.

**Result:**

Maintaining dignity was described when caregivers had the time and the will to see and listen to patient and had the courage to see what they did not want to see, allowing their inner powers to act with the purpose of doing good. In elderly care, it was important that elderly persons are involved as members of society and experience respect, confidence, security and charity. Indignity was described when caregivers did not allow patients to have their will and when they had unethical attitudes, ignoring patients and creating powerlessness. The feeling of being abandoned and not being taken seriously are also described in elderly care.

**Conclusion:**

Findings show how caregivers fulfil their ethical responsibility by seeing, listening and being a part of the time and place. The will to do good includes the courage to preserve dignity and human value rests on being created as a human being. More research is needed about ethical and moral responsibility in clinical practice.

## Introduction

This study summarises 28 empirical studies of how human dignity can be achieved in caring science research. We want to get a broader understanding of the differences and similarities of human dignity across different clinical practice. Caring science is an autonomous knowledge discipline based on an ethical perspective with the research interest directed at patients, families and healthcare professionals (1, 2). The human sciences recognise the dignity of the other on an ontological level (2). Dignity is a central and complex phenomenon in caring (2). Dignity is based on source of values and is expressed as absolute and relative dignity. The values are holiness, freedom, responsibility, duty and serving one's fellow men. A person's health and well‐being are essential to living as full a life as possible. There is a flexibility at preserving the experience dignity influenced by culture (2). Eriksson proposes that through a caring communion human dignity is born. The ethical ideal is based on the idea of human dignity and is rooted in a personal ethos that illuminates and indicates the direction of the professional nursing care (3). Ethics will always produce more questions than satisfactory answers, and in this study, we start from an ethic that touches the deepest core of care and understanding of human rights to preserved dignity (1, 2, 3, 4). According to ICN (5), nurses have the responsibility to protect patient dignity and there is a legal and ethical duty to take care of the patient as much as possible. The four basic responsibilities are as follows: promoting health, preventing illness, restoring health and alleviating suffering (5). According to Blomberg, Bisholt and Lindwall (6), nurses and health professionals have both an external formal and an internal personal ethical responsibility in nursing care. The professional caregiver's ethical responsibility and moral responsibility is to protect the unique person's dignity from vulnerability, humiliation and violations. If the dignity of the patient is not protected, the patient is subject to unnecessary and perhaps unbearable suffering (6). Caregivers who have witnessed or participated in undignified care often experience an internal conflict of values, one becomes conflicted with oneself and one's ethical values (7). Caring includes taking responsibility for the ethical dimension including respect for the unique person. Respect for human dignity is crucial to how the patient experiences care. When respect is excluded, there is a risk that the patient experiences suffering of care (3, 4). Responsibility lies in understanding the human right to dignity and being care for with respect (8). According to Arman (9), suffering of care can also be understood as indignity. The ethics of the caregiver are evident in every meeting between the patient and the caregiver regardless of the time, space and place of the meeting. The caregiver consciously or unconsciously chooses his or her ethical approach to take responsibility for the patient's care. In ethical care, the patient is seen as a unique person of value (10, 11). The dignity of the human being is reflected in the caregiver’s attitude and how he/she confirms his/her own dignity, as well as the dignity of relatives and caregivers (12). Although most caregivers are prepared to uphold the ethical ideal, the mindset of love and the ethical principles that are rooted in the idea of human dignity, violations of human dignity constitute the most prominent ethical phenomenon in practice. Ethics is evident in the caregiver’s choice of actions and activities that aim to preserve human dignity. We understand human dignity as central to care at all practice, but being an intrinsic and abstract concept, it might be difficult for healthcare professionals to have a shared understanding of how to apply dignity in clinical practice.

## Aim

*The aim was* to summarise studies on human dignity to gain a deeper understanding of how it can be achieved in caring science research and to gain a broader understanding of the differences and similarities across the different caring contexts. The aim was also to gain a broader understanding of the differences and similarities of human dignity across different clinical practice. The research question was as follows: *How do patients, nurses, health care professionals and next of kin experience human dignity in clinical practice?*

## Method

This systematic literature review was inspired by Morse (13) and reduces research into key findings in a reliable way and offers a means of enabling caring professions to keep abreast of research (14). This approach was useful when the topic lends itself to theoretical and qualitative methods of investigation. The review process involved five stages: (a) literature search, (b) data evaluation, (c) data analysis, (d) reporting and (e) conclusion.

The first stage, (a) literature search, was conducted with repetitive searches in the MEDLINE, Scopus, PsycINFO, CINAHL and Web of Science databases using the words ‘human dignity’. The searches were performed from March through July 2019.

In the second stage, (b) data evaluation, we search for scientific studies from different clinical practice. Inclusion criteria were as follows: caring context, patient/person experience and interpretation of human dignity, caregivers' experience and interpretation of human dignity in different clinician context and next of kin experience of human dignity in elderly care. Exclusions criteria were, not caring context, children’s experience of human dignity and books or posters. More than 800 articles were found with the topic of human dignity and with caring science we found over 400 studies. Finally, we decided to take our starting point in our own 28 empirical studies and make a re‐analysis of these studies to uncover the phenomena human dignity in different clinical practice.

In the third stage (c), these 28 studies involved clarifying and modifying the concept human dignity from a caring science perspective and were from four different clinical practice. Sample in the studies was nurses and healthcare professionals (n = 115), students (n = 109), next of kin (28) and elderly patients (28) make up a total of 280 informants. Through this process, we hoped to gain a deeper understanding of how experience of human dignity can be achieved in caring science research. The included 28 studies use qualitative designs, and several have a hermeneutical philosophical approach. The data in the various studies have mainly been collected with the help of interviews and critical incident technology (14, 15). Critical incident technology is description of situations that participants themselves have experienced. To be critical, the referenced event must have been crucial to the experience of success or failure in care work (15). In some studies, participants were asked to written one positive and one negative situation where they experienced preserved or humiliation of human dignity in clinical practice. Some studies use hermeneutic text interpretation and qualitative content analysis (16). The result is synthesised and constructed by an inductive methodology. According to the Morse review model (13), the data analysis strategy was conducted in a systematic and critical way (14).

In the last two stages (d) and (e), the result was performed and reported with a conclusion. All 28 studies are published in international journal and carried out in Nordic countries. In Table 1, we comprise the information on the studies' authors, publication year, country, design, aim, sample, data collection and analysis.

**Table 1:** Summary of the reviewed studies into human dignity

Authors, year, country (References)	Research design	Aims and objectives	Sample	Data collection Analysis/interpretation
Sundell et al. (2010) (18) Sweden	Qualitative design Hermeneutic approach	To capture and describe what older patients undergoing the surgical process wished to talk about during perioperative dialogue’s with nursing staff	Nurse anaesthetist (n = 6) operating theatre nurses (n = 3) documented perioperative dialogues with 42 older patients	The text was analysed using hermeneutic text interpretation
Lindwall et al. (2010) (19) Sweden	Qualitative design Hermeneutic approach	To described who the older patient undergoing surgery is as a part of the perioperative dialogue and in order to understand their needs	Nurse anaesthetist (n = 6) operating theatre nurses (n = 3) documented perioperative dialogues with 54 older patients	The text was analysed using hermeneutic text interpretation
Blomberg et al. (2015) (20) Sweden	Qualitative design Hermeneutic approach	To present what OTN students experienced and interpreted as preserved dignity in perioperative practice	Specialist OTN students (n = 60)	The text was analysed using hermeneutic text interpretation
Willassen et al. (2015) (21) Norway	Qualitative design Hermeneutic approach	To present what OTN students experienced and interpreted as undignified caring and dignity in perioperative practice	Specialist OTN students (n = 60)	The text was analysed using hermeneutic text interpretation
Taraldsen Valeberg et al. (2017) (22) Norway	Qualitative design Hermeneutic approach	To describe how nurse anaesthetists’ students experienced patients’ dignity in perioperative practice	Specialist NAS student (n = 23) write critical incidents and do interpretation on preserved and violation of human dignity	The text was analysed using hermeneutic text interpretation
Vendlegård et al. (2010) (23) Sweden	Qualitative design Hermeneutic approach	To describe how nurses’ experiences preserved dignity versus violated dignity in surgical practice	Nurse (n = 11), unrolled nurses (n = 3) fysioterapeut (n = 1) total n = 15 Between 28 and 58 years and 2–15 years of experience in surgical nursing care	Critical incident technique with 49 written stories were analysed using hermeneutic text interpretation
Lindwall et al. (2014) (24) Sweden	Qualitative design Hermeneutic approach	To obtain an understanding of what is experienced as human dignity by nurses in surgical practice	Surgical nurses (n = 11)	Critical incident technique with 49 written positive and negative stories were analysed using hermeneutic text interpretation
Bredenhof Heijkenskjöld et al. (2010) (25) Sweden	Qualitative design Hermeneutic approach	To understand how nurses experienced patients' dignity in Swedish medical wards	Medical nurses (n = 12) 2–43 years experience	Critical incident technique with 45 written positive and negative stories were analysed using hermeneutic text interpretation
Abelsson et al. (2017) (26) Sweden	Qualitative design	To describe what specialist ambulance nurse students experienced as preserved and humiliated dignity in prehospital emergency care	Specialist ambulance nurse SAN students (n = 26)	Data were 52 critical incidents Qualitative content analysis
Abelsson et al. (2018) (27) Sweden	Qualitative design	To describe specialist ambulance nurse students' experiences of ethical conflicts and dilemmas in prehospital emergency care	Specialist ambulance nurse SAN students (n = 24)	Data were 24 critical incidents All incidents were analysed using hermeneutic text interpretation
Gustafsson et al. (2013) (28) Sweden	Qualitative design Phenomenological–hermeneutic approach	To illuminate the meaning of maintenance of patient dignity in forensic care	Nurse and unrolled nurses (n = 7) between 30 and 52 years, and all have at least 2 years’ experience from forensic	Focus groups interviews during eleven‐month 2007. The text was analysed by phenomenological and hermeneutical method
Gustafsson et al. (2014) (29) Sweden	Qualitative design hermeneutic approach	To describe nurses’ experiences of violation of patient dignity in clinical caring situations in involuntary psychiatric hospital care	Nurses (n = 15) Between 27 and 52 years	Two interviews groups during 9 months The text was analysed and interpretation by Ricoeur and Gadamer
Lindwall et al. (2012) (30) Sweden	Qualitative design hermeneutic approach	To describe how nurses experienced incidents relating to patients dignity in a psychiatric nursing practice	Nurses (n = 16) between 26 and 54 years experienced between 2 and 10 years from different inpatient and outpatient psychiatric settings	Data were 77 critical incidents, both positive and negative incident was analysed with hermeneutic text interpretation
Gustafsson et al. (2017) (31) Sweden	Qualitative design hermeneutic approach	To describe how nurses experienced violations of their own dignity in psychiatric inpatient setting	Nurses (n = 15) and inpatient (n = 6).	Two different interview group during 9 months The text was analysed by hermeneutic text interpretation
Heggestad et al. (2015) (32) Norway	Qualitative design hermeneutic approach	To present and discuss some findings on how older people in nursing home experience dignity related to dependence and autonomy and how their dignity may be promoted.	Residents (n = 28) from six nursing homes between 62 and 103 years	Interviews with a research guide K value analysis method with three steps
Høy et al. (2016) (33) Denmark	Qualitative design	To illuminate the meaning of maintaining dignity from the perspective of older people living in nursing homes	Residents (n = 28) from six nursing homes between 62 and 103 years	Interviews with a research guide Data were analysed with a phenomenological–hermeneutical method
Sæteren et al. (2016) (34) Norway	Qualitative design	To answer the following question: What do the nursing home residents do themselves in order to maintain their dignity	Residents (n = 28) from six nursing homes between 62 and 103 years	Interviews with a research guide. The text was analysed and interpreted by hermeneutical approach
Slettebø et al. (2016) (35) Norway	Qualitative explorative and descriptive design	To examine how nursing home residents experience dignity through the provision of activities that foster meaning and joy in their daily life	Residents (n = 28) from six nursing homes between 62 and 103 years	Interviews with a research guide The text was analysed by content analysis
Nåden et al. (2013) (36) Norway	Qualitative explorative design	To gain further knowledge about how dignity is maintained, promoted and neglected in nursing home residents	Family caregivers (n = 28) between 47 and 85 years from six different nursing homes in Scandinavian	Interviews with a research guide The text was analysed and interpreted by hermeneutical approach
Lohne et al. (2014) (37) Norway	Qualitative design	To highlights narratives from perspective of family caregivers	Family caregivers (n = 28) between 47 and 85 years from six different nursing homes in Scandinavian	Interviews with a research guide The text was interpreted by a phenomenological–hermeneutical approach
Råholm et al. (2014) (38) Norway	Qualitative design	To describe different perspective of dignity in the care of residents experienced by family caregivers	Family caregivers (n = 28) between 47 and 85 years from six different nursing homes in Scandinavian	Interviews with a research guide The text was analysed with hermeneutical text interpretation
Caspari et al. (2014) (39) Norway	Qualitative hermeneutical design	To explore how residents in nursing homes experiences that their dignity is promoted and attended to	Family caregivers (n = 28) between 47 and 85 years from six different nursing homes in Scandinavian	Interviews with a research guide The text was analysed according to *K* value three levels of interpretation
Rehnsfeldt et al. (2014) (40) Sweden	Qualitative hermeneutical design	To investigates the individual variations of caring cultures in relation to dignity and how it is expressed in caring acts and ethical contexts	Family caregivers (n = 28) between 47 and 85 years from six different nursing homes in Scandinavian	Interviews with a research guide The text was analysed by hermeneutical interpretation
Lohne et al. (2017) (41) Norway	Qualitative design	To explore dignity as a core concept in caring, and how healthcare personnel focus on and foster dignity in nursing home residents	Healthcare personnel (n = 40) from six different nursing homes in Scandinavian	Interviews of six focus groups of health personal. A semi‐structured guides were used for three or five times with each group at each nursing home. The text was analysed by content analysis.
Lohne et al. (2010) (42) Norway	Qualitative design with a phenomenological–hermeneutic approach	To find out how persons suffering from multiple sclerosis experience and understand dignity and violation in the context of a rehabilitation ward	Patients with the diagnosis MS (n = 14) participated	Individual interviews. The hermeneutic analysis was performed inductively, the purpose being to extract meaningful content from the patients’ experiences.
Slettebø et al. (2009) (43) Norway	Qualitative design	To report of a study conducted to determine how people who suffer from head injuries perceive respect for their dignity and to discover what patients mean by the concept of ‘dignity’	Patients suffering from head injuries (n = 14) participated	Qualitative interviews The text was analysed with content analysis.
Lohne et al. (2016) (44) Norway	Qualitative hermeneutic design	To explore how the family caregivers experienced and narrated their daily life as caregivers for individuals suffering from multiple sclerosis	Family caregivers (n = 9) Between 34 and 73 years	Individual interviews with a guide. The text was interpreted by Ricoeur
Caspari et al. (2013) (45) Norway	Qualitative design	To explore how healthcare personnel comprehend the term dignity and what they do to attend to, preserve and promote the dignity of patients in the rehabilitation context	Staff (n = 9) from three different rehabilitation centres	Qualitative focus group interviews The text was analysed with content analysis.

### Ethical issues

Good ethical practice in preparing and publishing literature reviews was applied which means that the authors aimed for transparency, accuracy and avoidance of plagiarism (17). All studies had followed ethical guidelines.

## Result

The main findings emerged from four clinical contexts: *Dignity in acute practice, Dignity in psychiatric practice, Dignity in elderly care* and *Dignity in rehabilitation care*.

### Dignity in acute practice

The result from perioperative nursing care research (18, 19) shows that dignity is about safeguarding and preserving by an ideal model the perioperative dialogue. From the patients’ point of view, the result shows that pre‐, intra‐ and postoperative dialogues create continuity and that professional nurse anaesthetist and operating room nurses are able to safeguard and preserve patient dignity. One study (18) states that elderly patients experienced that the continuity of the perioperative dialogue protects patient dignity. The patient described that they were given the opportunity to understand what was happening in and through his/her body. In this perioperative dialogue, it was possible to relieve patient suffering and to be a part of the operating process. The patient felt in safe hands during the conversations (19). Nurse anaesthetist and operating room nurses, who together with the patient participated in the perioperative dialogue, took responsibility for continuity being created and that the patient's dignity was maintained in the perioperative nursing care practice (18, 19).

According to Blomberg et al. (20), operating room nurses (OTNs) protected the patient's dignity through their presence in the operating room. Dignified care is associated with community and reciprocity where the patient and caregiver interact and challenge the care. The feeling of being outside the caring relationship can create suffering (20). Caregivers who behave unprofessionally expose the patient’s body, see the patient as an object, practice care that leads to suffering and create humiliation of dignity (21). When OTN students witness situations that they interpret as undignified care, courage and the will to not abandon the patient are required. In another study (22), taken from the perspective of students in anaesthesia care, violated dignity appears when caregivers ignore the patient, talk over the patient’s head and do not respect the personal sphere. In perioperative practice, human dignity appears in the will not to abandon the patient. Violated dignity appears to not be seen and not to show consideration for a vulnerable body.

In surgical practice, nurses experienced that patient dignity was preserved and violated. A study (23) revealed that several healthcare professionals experienced that patients, both old or young who become acutely ill or must undergo a planned operation or who are at the end of life, expect caregivers to maintain their dignity. The study shows that human dignity was preserved when the health professionals allowed the patient to tell their story and they listened even if it was filled with discomfort, pain and suffering. The result also shows that health professionals violated patient dignity when they behaved rudely towards the patient, treated the patient as if they were invisible and humiliated the patient in palliative care. According to Lindwall and von Post (24), caregivers want to preserve patient dignity when the caregiver dares to come close to the patient and make the patient visible. To preserve the patient's dignity, a permissive atmosphere in the room is required for the patient to dare to trust the caregiver. In surgical practice (23, 24), human dignity is based on two aspects. The first is preserved dignity – when caregivers have the time and the will to listen to and see the patient and have the courage to see what they do not want to see and allow their inner powers to act with the purpose of doing good. The second is violating dignity – when the caregiver chooses not to see, listen or show respect to the patient, the suffering person.

Other studies in acute practice have been done in medical practice and prehospital practice. In medical practice, one study (25) shows that the patient has an expectation of being treated with respect. When the patient and the caregiver see each other, the caregiver becomes ethically responsible for the patient being seen as a friend and a fellow human being. Patient dignity is preserved when the patient can speak about themselves and their life. The results also show that patients express that they want to be involved in their care and that nurses dedicate personal time. In the same study, experiences of violated dignity emerged when caregivers did not respect patient will or when nurses abandoned the patient, neglected the patient, did not believe what the patient said and did not take the patient's situation seriously. Healthcare professionals created a feeling of powerlessness that led to dignity being violated. The result from a medical ward shows that human dignity was shown by caregivers seeing the patient as a friend, allowing the patient to talk and inviting them to be involved in their own care. However, when caregivers do not respect the patients, when they do not allow the patient to have their own will and have an unethical attitude, patient dignity is experienced as violated dignity (25).

Two studies from prehospital practice (26, 27), where students participated in research illuminating how dignity was shown to the patient, showed how human dignity can be preserved by the ambulance nurse being there for the patient (26). Students saw how the ambulance nurses met the patient and respected the patient's need for help and the desire for the body to be protected from other people's eyes. How the ambulance nurse is by the patient’s side and monitors the patient during transport has a bearing on how the patient experiences their dignity. Humiliated dignity was described through abandoning, disrespecting and ignoring the patient (26). In another study from the student’s perspective in prehospital practice (27), the ambulance nurse experiences ethical dilemmas and conflicts of values when they witness how others violate patient dignity, and students see how caregivers do not safeguard the patient’s body or identity. Healthcare professionals got into conflicts when the patient was not treated in an ethical manner, and seeing how caregivers put themselves in a power position is described as an ethical dilemma by students when they choose not to intervene (27). In prehospital practice, human dignity was preserved when caregivers respected and protected the patient. Humiliated dignity occurs when caregivers ignore and do not protect the patient. Ethical dilemmas occur when caregivers do not safeguard the patient’s body or do not treat it in an ethical manner.

### Dignity in psychiatric practice

Four empirical studies (28, 29, 30, 31) described human dignity in psychiatric practice. One study shows (28) that the maintenance of patient dignity in clinical caring situations in forensic care means that the nurse through the caring relation acts with a sense of protection and respect. Protecting the patient could be described as confirming to the patient that he/she has the same rights as others. Respecting the patient could be about teaching the patient to create respect and taking the patient’s expressions seriously. Meeting the patient with dignity was expressed as walking the extra mile and doing that little bit extra. This study illuminates‐maintained dignity in a forensic care practice, and it is important to be the patient's guardian as well as to protect the patient from himself and others (28).

Caregivers in psychiatric practice (29) care for people with psychiatric illnesses. The psychiatric care can be open or closed and the care is done voluntarily or under duress. In this study, undignified care was studied. The nurses experience situations where the patients were not taken seriously by staff when patients complained or tried to express physical symptoms or pain in the body. Only referring to the mental illness can have fatal consequences for the patient as well as ignoring and not involving the patient in their care. During involuntary psychiatric hospital care, the patient can be betrayed in many different ways and this is always at the cost of the patient’s human dignity (29). The results of the study (30) show how mental health caregivers in psychiatric nursing practice saw patient dignity being preserved when caregivers had the courage to be present. However, they also saw offended dignity when caregivers created powerlessness. Maintaining patient dignity could be achieved by meeting the patient problems and needs that he/she expresses, and seeing the unspoken wish to be listened to. Indignity was experienced by the caregiver as a feeling of powerlessness such as when other caregivers exerted their power over the patient, punished the patient in different ways or neglected the patient when waiting. Another study highlights dignity as an important concern both for caregivers and for patients in psychiatric inpatient practice (31). Experiences of violations of their own dignity as nurses are described when other caregivers treat them as subordinates, when they are left in the lurch by colleagues or when forced to perform repulsive acts. The results also outline situations where patients violate nurse dignity by calling nurses insulting names or by physically violating nurses (31).

Research in psychiatric practice shows the importance of caregivers engaging personally, valuing themselves and their care to create genuine encounters with the patient. Caring in this context is a challenge as caring involves protecting the patient's dignity, showing respect for the patient as a human being, and being a fellow human. Caregivers who have chosen to safeguard patient dignity may end up in a conflict of values, when they find that other caregivers act unethically.

### Dignity in elderly care

Dignity in elderly care is in focus on ten studies (32, 33, 34, 35, 36, 37, 38, 39, 40, 41). Nursing homes are supposed to be a home but some are a crossroads for vulnerable individuals as well as being a power system. Maintaining the human dignity in nursing homes and at the end of life is a key objective in elderly care. It is an important ambition to enhance feelings of dignity and worthiness for every patient within elderly care. Some elderly person has disabilities, various problems and the need for care and treatment, which is why the reason for placement in a care home varies greatly. These persons have a reduced opportunity to 'be able to manage themselves' and as a result become involuntarily dependent on professionals in elderly care. Professional caregivers strive to see each person as the carrier of their own intrinsic value. It may seem obvious that elderly people should be able to maintain their dignity and live a life based on their ability, identity and personality. In Heggestad et al. (32), the concept of dignity is related to living in nursing homes and autonomy. An institution's organisational framework influences people's experiences of independence and dignity. As time and resources increase or decrease, independence and opportunities for the elderly are affected by living the life they want to live. The results show that negative views about dependence, institutional frames and structures in the nursing homes and the attitudes and actions of healthcare personal may diminish independence and lead to a lack of autonomy. Maintaining dignity is shown to be closely connected to the feeling of freedom and one's own autonomy and independence.

In another study (33) from the perspective of residents, maintaining dignity was constituted in a sense of vulnerability to self and elucidated in three forms: being involved as a human being, being involved as the person one is and striving to become and being involved as an integrated member of society. Moving to a nursing home is an assistance that challenges a person's ability to adapt to a new situation in life. In order to experience health and dignity as elderly person, it is important to expand their inner space. Residents (34) tried to expand their life space, both physical and ontological in order to experience health and dignity. The person's own healthcare resources are of great importance for their ability to experience a life of dignity and reciprocity, a sense of a meaningful life. In Slettebø et al. study (35), from the perspective of the residents, activities that fostered experiences of dignity were highlighted. The activities were based on human life experiences and provided meaning and joy in the lives of the residents. Dignity can be demonstrated by seeing the elderly person and being interested in the person and being present in the elderly person's life. Presence means being close in time and space, allowing past and present time to meet, and sharing thoughts that the elderly have about the future. Based on relatives' stories, dignity is about not feeling abandoned and left alone (35).

From the family caregiver’s perspective, elderly loneliness (36) reveals the vulnerability of the person. Many relatives described varieties of indignity in care and they felt concern for their loved ones, a form of suffering. A feeling of being abandoned is described as a feeling of not belonging, deprived of dignity due to acts of omission, deprived of confirmation and deprived of parts of life. Family caregivers’ experiences in nursing homes (37) show that one should treat others as one would like others to treat you. Dignity was maintained in experiences of respect, confidence, security and charity. Uneasiness occurred when indignity arose. In Råholm et al. (38), human dignity can be understood on three levels: concrete, relational and existential. Dignity affects both residents and their relatives, and it takes trust for the elderly patient to tell his/her story and surrender in the hands of the caregiver. When relatives describe the weakness and vulnerability of residents, dignity is understood at an existential level.

Dignity from the relatives’ perspective concerned what was deemed important to the resident according to his/her existential needs and concerns. Comfort depended on having a homely and practical room; having close contact with family, friends and staff; and having aesthetic needs attended to as well as ethical, cultural and spiritual needs being met (39). However, human dignity is shown in the study to be often challenged in the course of daily activities. Preserved dignity is 'doing the little extra', where small things can make all the difference. The results (40) also show that relatives see meetings with caregivers as important.

In Lohne et al. (41) study, dignity from the caregivers’ perspective illustrates that dignity emerges when elderly feel independent, feel a part of their daily lives, have influence and have the right to make their own decisions about their lives. Human dignity shows itself in the elderly care practice as being confirmed, both as a resident and as a relative. Dignified care is based on mutual trust, security, integrity, respect and kindness. Humiliations are experienced as loneliness and a feeling of being abandoned. The experience of abandonment can be specifically related to being left alone in their rooms or sitting for long periods at a time in a common room. It is important for them to be taken seriously. Situations that maintain or humiliate dignity are described in several studies. Dignity was maintained in situations where respect, trust, safety and friendliness were demonstrated in accordance with the international code of nursing ethics. However, patients became perturbed when their dignity was violated. This led to conflicts between patients’ families and nursing home staff.

### Dignity in rehabilitation care

Dignity in rehabilitation care was in focus on four empirical studies. In this context, focus was on patients struggling with multiple sclerosis (41), patients struggling after head injuries (42), family caregivers to patients struggling with multiple sclerosis (43) and healthcare personnel struggling with preserving and promoting dignity within the rehabilitation practice (44). According to patients suffering from multiple sclerosis, their struggling was described as invisibility and as being captured by fatigue (41). The challenging experience of the continuing requirement of fighting for oneself was expressed as the one who does not, will not, receive, in the context of fatigue. The battle against decreased mobility due to MS and the increasing fatigue made the participants exhausted and full of despair. Despite this, none of the participants gave up (41). Another study focussing on patients suffering from head injuries (42) narrated that the meaning of dignity was closely connected to a feeling of self‐management which again resulted in meaningfulness. Being respected by nurses as a person was appreciated and was understood as being heard, being given time and being respected. Dignity gave a feeling of growing and that life had a meaning and challenges. Every improvement gave confidence and competence to the patients. At the same time, one said, *the better I get, the worse I feel –* meaning that the better he became, the more he understood how injured he was. The participants admitted that hiding symptoms was a way of preserving dignity. According to the participants (42), violation of dignity occurred when they felt like a tennis ball in the system or when they lacked information from healthcare professionals. According to Lohne et al. (43), the struggle of invisible caregivers of patients suffering from MS concerned experiences of feeling uneasy on behalf of their family member. At the same time, their social networks gradually disappeared when they needed them most. Waiting for help, answers and results became a new lifestyle, and finances were put under strain. Uncertainty, vulnerability and bitterness overwhelmed the family as the symptoms progressed. They lived a daily struggle in silence and invisibility. At the same time, they were busy and exhausted. They felt alone with the daily responsibility and had to give up their own lives. According to healthcare professionals in a rehabilitation practice (44), they considered that dignity was promoted and preserved when patients became active in the process of rehabilitation, when the staff members were able to cope with the patient’s disability, and when patient feelings and thoughts were respected. The staff never used the word dignity, rather they used expressions such as quality of life, meaning helping the patients to feel more dignified and worthy. At the same time, the healthcare professionals were aware of the importance ofpreserving and confirming patient resources. They also stressed the importance of seeing and listening and sustaining patient autonomy described in terms of seeing the person and not only the diagnosis (45).

## Essence

Human dignity is central in caring science. Dignity, an ethical and moral dimension in clinical practice, is experienced by patients/residents, nurses, healthcare professionals and next of kin/family caregivers. In this research, dignity is about having a value as a human being and that professionals have an ethical attitude that means showing respect and taking responsibility for the other. The nurse has both a formal external responsibility and an internal personal responsibility for the care to rest on a scientific foundation. Within different nursing care cultures, there are reorganisations, new directives are formulated and there is a constant development of different treatment strategies. The demands for increased productivity and efficiency in health care appear to be more important than developing knowledge of caring ethics and dignity. Ethics teaches us as healthcare professionals to become moral responsibility, aware to see, to listen and to feel and to act following an ethical compass and to allow ethos to be the guide. Eriksson writes (4) that it is in the light of human dignity and her ethos that the substance is formed in each individual caregiver's act and the good, probable and comfortable form of care is present. Dignity is contested and debate is likely to continue. In these 28 empirical studies, some foci have been on elderly people in acute and in nursing homes who can be particularly vulnerable, and it is essential that extra attention is given to making sure that healthcare professionals care for them with dignity.

## Discussion and reflection

This study based on 28 empirical studies from four different clinical practice in Nordic countries describes how human dignity manifests itself in clinical practice. By using the hermeneutical philosophy from Gadamer (46), we present the results of previous research with a focus on human dignity, and a new understanding is revealed of how vulnerable human dignity is in various clinical care practices. The importance of the patient being allowed to be the suffering person (3) in the care as well the importance of the caregiver watching and protecting his/ her dignity in meetings and the caring relationship is also clarified. Through individual and group interviews, written stories and both positive and negative critical events, new knowledge has emerged about human dignity in practice. The studies are from different practice, and the results may be partly seen as general and relevant in all health care. But in this presentation, the texts are seen as unique to each practice. In meetings between the patient and the caregiver, there are ethical obligations to preserve patient dignity (2, 4). This relationship is surrounded by the dignity of both, and the professional nurse's ethics (5), interest and willingness to do good for the suffering person (9, 10, 11). The results of our studies can be summarised as being that dignity is about having a value as a unique person where dignity is experienced by being seen, listened to and believed in. Dignity also means being treated with respect and reverence in a caring community. Showing respect for the other means allowing the individual to be unique, having his/her own needs and desires taken seriously. Dignity is a constant presence that involves taking responsibility (8) for the other and having the courage to intervene and prevent the patient's dignity from being violated so that the patient falls into a medical condition. Lévinas (47) argues that responsibility ethics means that one is not free in relation to the other and that there is something infinite in the ethical requirement. Regardless of illness and diagnosis, there is the will of professional caregivers within the chosen clinical care practice to preserve the patient's dignity (48). Some studies describe clinical situations interpreted by caregivers as uncaring, violations. The violations are seen as deviations from the ideal, the good care. In situations where experiences of dignity can no longer be realised, threats of violation of human dignity arise. There are healthcare professionals who experience internal conflicts of values when they witness how other caregivers violate patient dignity. The will to do good includes the courage to intervene and prevent the degradation of the dignity of patients or caregivers. In the meeting between patient and nurses have the obligation to maintain the patient's dignity by alleviating suffering (3). This relationship is surrounded by the dignity of both and the professional ethics, the interest and the will to do good, includes the will to be there for the suffering person (3, 9). The caregiver helps the patient increase his/her value by sharing the suffering, being absorbed by the other person's suffering and being compassionate (49). Students express the importance of becoming more aware of moral, ethics and dignity and that patients are mostly cared for based on thoughts of a good approach to maintaining the dignity of others. In our research, we assumed that there is healthcare professional (physicians, Registered Nurses, enrolled nurses and mental health caregivers) who maintain or violate the dignity of the patient and other professionals. The results can be summarised that maintaining dignity is about caregivers having the ability to create time and space, being present in the meeting and contributing to a calm atmosphere. Showing respect for the other allows the individual to be unique, to take his/her problems and needs and desires very seriously and to have the courage to intervene and prevent the violation of the patient's dignity and thereby prevent suffering as a result of caregiving. The results also show events that are interpreted as violations, and this is seen as deviations from all the ideals of good care. There are caregivers who experience internal conflicts of values when they witness how other caregivers violate patient dignity. Ethics teaches us as to act following an ethical compass. We believe this research has improved human dignity in clinical practice through increasing awareness on patients’ needs for dignity. Furthermore, we hope that reflections on dignity in praxis and the various aspects of dignity presented in this study aid in supporting and caring for patients needs connected to dignity.

## Strengths and Limitations

This literature review has some limitations. Firstly, the number of studies is limited, although specific inclusion criteria were used in order to get a deeper understanding of human dignity as it appears in different clinical practice. The second limitation concerned the study design. This review only included research where the authors had been responsible.

## Conclusions

This study result shows how patients/residents, nurses, healthcare professionals and next of kin experience human dignity in clinical practice. Healthcare professionals fulfil their ethical and moral responsibility by seeing, listening and being a part of the time and place. Human value rests on being created as a human being and holding the human office. Therefore, all people have equal value and have an absolute and relative dignity. Eriksson (4) further claims that human freedom is its dignity, while being bound to the responsibility of being human. In connection with respect and the responsibility, she refers to the mantra of ethics; I was there, saw, testified and became responsible.

## Ethical approval

Principles of research ethics were strictly observed throughout the study. No formal ethical approval was needed or sought for this study.

## Funding

This article rests upon empirical research that was funded by the Research Council of Norway (grant number 190889/V50).

## Conflict of interests

The authors report no conflict of interests and are responsible for the content and writing of the paper. The author declares that they have no competing interest.

## Author contributions

LL and VL participated in the design of the project and in data analysis and interpretation. LL was responsible for drafting the manuscript. Both authors have read and accepted submission to the journal.
